# Compositional variances in petal cuticular wax of eight rose species and their impacts on vase life under water-loss stress

**DOI:** 10.3389/fpls.2024.1412617

**Published:** 2024-09-05

**Authors:** Xuan Hao, Junheng Lv, Zixian Zhao, Yuxin Tong, Minghua Deng, Jinfen Wen

**Affiliations:** ^1^ Faculty of Architecture and City Planning, Kunming University of Science and Technology, Kunming, Yunnan, China; ^2^ College of Landscape and Horticulture, Yunnan Agricultural University, Kunming, China

**Keywords:** rose petals, cuticle waxy, water loss stress, vase life, alkanes

## Abstract

Cuticular wax is the first barrier between plants and the environment. Here, the densities of cuticular wax crystals on the petals of eight rose cultivars were determined to be sparse; the crystals were mostly granular and only a few rod-shaped crystals were observed in ‘Sweet’. The total contents and chemical compositions of waxes were significantly different among the rose varieties. The waxes were mainly composed of n-alkanes, iso-alananes and alkenes. Under water-loss stress, ‘Diana’ and ‘Carola’ cultivars, having high petal wax contents, had low water permeability levels, long vase lives, high relative water contents and low relative conductivity levels. However, the low wax contents of the ‘Jubilance’ and ‘Candy Avalanche’ cultivars resulted in high water permeability levels and short vase lives. Pearson correlation analyses showed the total wax content in petal epidermis was positively correlated with vase life. The data provide novel insights into the compositional variances in the cuticular waxes of rose petals and their impacts on cut rose vase lives.

## 1 Introduction

Terrestrial plants are covered with a layer of cuticle wax, which evolved approximately 450 million years ago. Both lower plants, such as mosses and ferns, and higher plants, such as gymnosperms and angiosperms, have above-ground plant surfaces that are covered with wax. The presence of this waxy layer plays important roles in terrestrial plant interactions with the outside world (Jetter et al., 2006). They form the interface between plants and the outside environment, acting as protective barriers (Riederer and Schreiber, 2001) against non-stomatal water loss (Premachandra et al., 1992), different abiotic stresses, such as excessive solar and ultraviolet (UV) radiation, cold and drought (Jenks et al., 1994; Long et al., 2003; Mamrutha et al., 2010; Yang et al., 2011), and bacterial and fungal pathogens and insects (Jenks et al., 1994; Eigenbrode and Espelie, 1995).

The carbon chains of plant cuticular waxes mostly contain between 20 and 34 carbon atoms; thus, consisting mainly of very long-chain fatty acids (VLCFAs, ≥ C20) and their derivatives. In addition, aldehydes, alkanes, ketones, primary and secondary alcohol, and alkyl esters, branched alkanes, alkenes, triterpenes, sterols and polyketones are included in the waxes of some plant species (Riederer and Schreiber, 2001; Kunst and Samuels, 2003; Jetter et al., 2006; Pollard et al., 2008; Bernard and Joubès, 2013; Lee and Suh, 2015a). The composition contributes to the morphology and hydrophobicity of the wax crystal (Mamrutha et al., 2010; 2017).

Plant cuticular waxes are divided into inner and outer. The inner waxes usually have no fixed shape, whereas the outer waxes can self-assemble to form unique wax crystal structures that are secreted onto the plant surface (Jetter and Schaffer, 2001). Barthlott and Theisen (1998) observed the morphology of cuticular wax crystals from more than 13,000 plants using scanning electron microscopy. They classified the wax crystal structures into 29 types, including filamentous, rod-shaped, tubular, sheet-shaped, plate-shaped, umbrella-shaped, spiral-shaped, cylindrical, dendritic and other morphological structures. Among them, tubular and sheet-shaped are the most common. The main types of fruit wax are amorphous film, plate/platelet, rod/stick and tubular (Wu et al., 2023). Different chemical compositions of waxes lead to different structures. Koch and Ensikat (2008) concluded that alkanes form smooth, membranous wax layers with no crystal structure, secondary alcohols or diketones mostly form tubular structures and primary alcohols mostly form lamellar structures.

Studies on plant cuticular waxes have mainly focused on leaf cuticle waxes of crops, such as barley (Sutanni et al., 2023), alfalfa and tomato (Wu et al., 2017), as well as lotus (Zheng and Li, 2023), and of fruit cuticle waxes from plants, such as litchi, apple (Dan et al., 2022; Huang et al., 2023), pear (Mao et al., 2022) and citrus (Wang et al., 2014). Studies in lily and rose showed that the contents of wax in the petal cuticle varied with different varieties (Cheng et al., 2019; Zhao et al., 2023). The petal cuticular wax of Snapdragon increased significantly from flower opening to senescence (Goodwin et al., 2003). In contrast, the amount of wax accumulation in inner and outer tepal of lily decreased significantly from green bud to open flower, and the content of the main wax component (n-) alkanes in outer tepal was significantly higher than that in inner tepal (Cheng et al., 2021). The vase life of the cut flowers is impacted by rapid water loss, cuticle wax is the main barrier to the uncontrolled water loss of aerial plant organs. A comparative study of five lily varieties demonstrated that varieties with high wax contents in petal epidermal had low water permeability (Zhao et al., 2023).

As one of the top four cut flowers in the world, rose flower’s water loss resistance is particularly important. However, the contribution of the petal cuticular wax content and components to the vase life has not been researched comprehensively in rose. In this study, eight rose varieties are employed to study the crystal structure, component and content of petal cuticular wax, as well as to analyze the correlation between petal cuticular wax content and vase life. This work lays a foundation for the breeding of roses in the vase life period.

## 2 Materials and methods

### 2.1 Materials and the dehydration-stress treatment

We used eight varieties of roses, ‘Carola’, ‘Jubilance’, ‘King’s Day’, ‘Candy Avalanche’, ‘Mount Shasta’, ‘Sweet’, ‘Diana’ and ‘Royal Highness’.

For the water-loss stress treatment, rose flowers harvested from the Kunming suburban greenhouse (Kunming China) in just full-bloom were cut, leaving a 5-cm stem, was placed in distilled water. In the control group, there was only distilled water. Cut flowers were then placed in an artificial climate chamber (23°C, 14-/10-h light/dark, 400 μmol m^–2^s^–1^ luminous flux density, 50%–60% relative humidity). A petal was taken for the determination of relative water content and relative conductivity at a fixed time every day, and the vase life was determined.

### 2.2 Scanning petals cuticular wax by electron microscopy

Each petal was washed with double-distilled water, the petal surface water was absorbed and then, each petal was fixed on the filter paper with a paperclip and dried in a drying box at 50°C. Each dried petal was cut into 5-mm × 5-mm pieces (avoiding the main vein) and plated with gold for 90 s using ion sputtering. The cuticular wax crystal structure was observed using a scanning electron microscope (TESCAN VEGA3, Brno City, Czech Republic) at 20 kV.

### 2.3 Analysis of wax components using gas chromatography and mass spectrometry

From flowers that were just in full bloom, petals were selected and washed with double-distilled water. After removing the water on each petal’s surface, the middle part of the petal (0.5 g) was taken and cut into pieces. Then, 5 ml of n-hexane was added three times to the pieces. The solution was transferred to a new tube, 4 mL of a C18 internal standard was added to the solution, and then the tube was shaken. The solution was dried using a 50°C-nitrogen blower HS2200 (Kuan Son, Shanghai, China), and then, 50-mL of derivative was added. The samples were dried in a 100°C-oven for 30 min, dried using a nitrogen blower, and then the proper volume of N-hexane was added. The chromatographic conditions were as follows: injection port at 300°C, splitless inlet sample, 1 mL min^−1^ carrier gas load flow rate, and temperature program of 80°C for 2 min, 10°C min^−1^ to 270°C min^−1^, holding for 2 min, 2°C min^−1^ to 310°C min^−1^, and holding for 1 min. The mass spectrometry conditions were as follows: transfer line at 320°C, ion source at 330°C, EI source, ionization voltage 70 eV, solvent delay 10 min, and scanning range 40–600 amu (Zhao et al., 2023).

### 2.4 Determination of water permeability

Cut flowers were collected in full bloom, petals were taken and the stem was immediately sealed with paraffin to prevent the loss of water from the incision. All the samples were in a state of complete water saturation before the petal cuticle transpiration rate determination using the Burghardt and Riederer (2003) method. The changes in petal weights were recorded every 30 min for 8 h. The transpiration rate was calculated from the change in the weight of the sample (ΔW), the change in time (Δt) and the surface area (A) measured according to Zhong et al. (2021), as follows: $T=\frac{\Delta W}{\Delta t\times A}$ Water permeability (P, m·s^-1^) was calculated in accordance with Zhao et al. (2023) as follows: $P=\frac{\text{T}}{{\text{C}}_{\text{WV}}^{*}({\text{a}}_{\text{petal}}-{\text{a}}_{\text{air}})}$ ,

where ${\text{C}}_{\text{WV}}^{*}$ represents the water vapor saturation concentration at the actual petal temperature, a_air_ represents the water activity in air, and a_petal_ represents the water activity in the petal.

### 2.5 Determination of relative water content

The petals of cut flowers were rinsed with double-distilled water, the surface waters were removed and the fresh weights (Fw) were recorded. The petals were then immersed in distilled water at 4°C for 24 h, then, the surface moisture was removed and the saturated fresh weights (Tw) were determined. The samples were placed in an oven at 105°C for 15 min, dried at 80°C to a constant weight and dry weights (Dw) were measured. The relative water content was defined as follows: RWC = (Fw− Dw)/(Tw− Dw) × 100%.

### 2.6 Measurement of relative conductivity

In accordance with the method of Liu et al. (2022), the petal surfaces were washed with double-distilled water, and the surface moisture was removed. Then, 10 petal discs were created using a perforator and immersed in 10 mL of deionized water. After soaking for 12 h at room temperature, the electrical conductivity (R1) of the extract was measured using a DDBJ-350 portable conductivity meter (Shanghai, China). Then, they were heated in a boiling water bath for 30 min and then cooled to room temperature and shaken. The relative conductivity (R2) of the extract was determined again. The relative conductivity was defined as follows: REC=R1/R2×100%.

### 2.7 Data analysis

All the experiments were repeated three times. All the data were analyzed for variance using SPSS 18.0 software (SPSS Inc, Chicago, IL, USA). Duncan’s multi-range test was used to evaluate the differences among the treatments. 0.05 was used to indicate the significant difference for the mean among the treatments.

## 3 Results and analysis

### 3.1 Observation of wax crystals on petals using scanning electron microscopy

**Figure 1:**
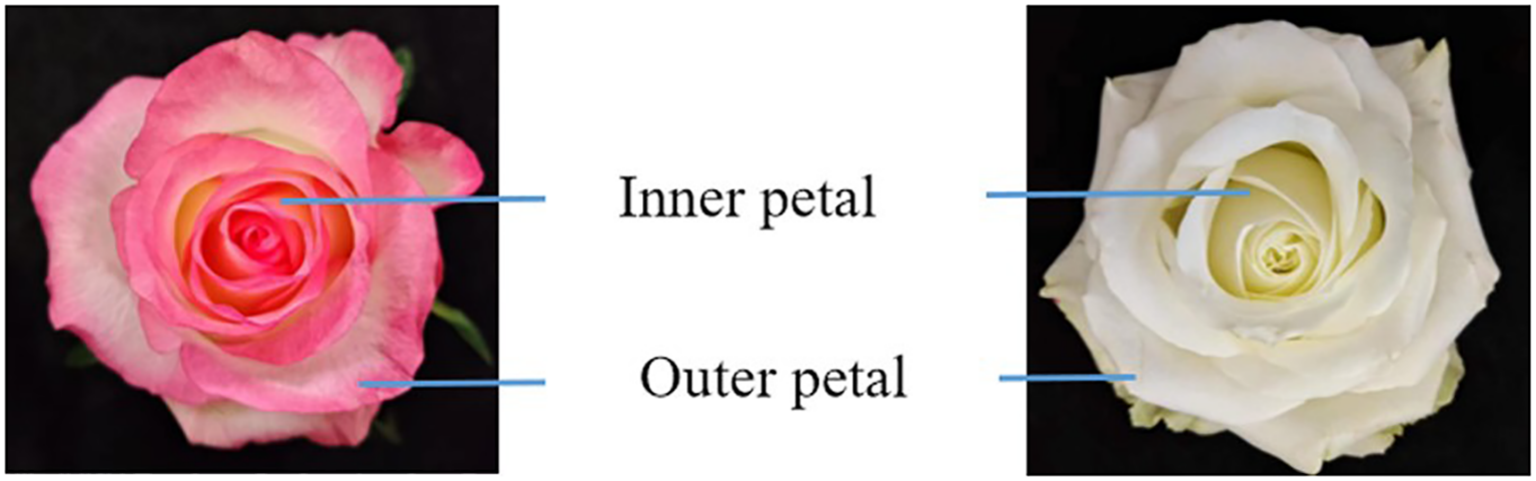
Inner petals and out petals of rose.

**Figure 2:**
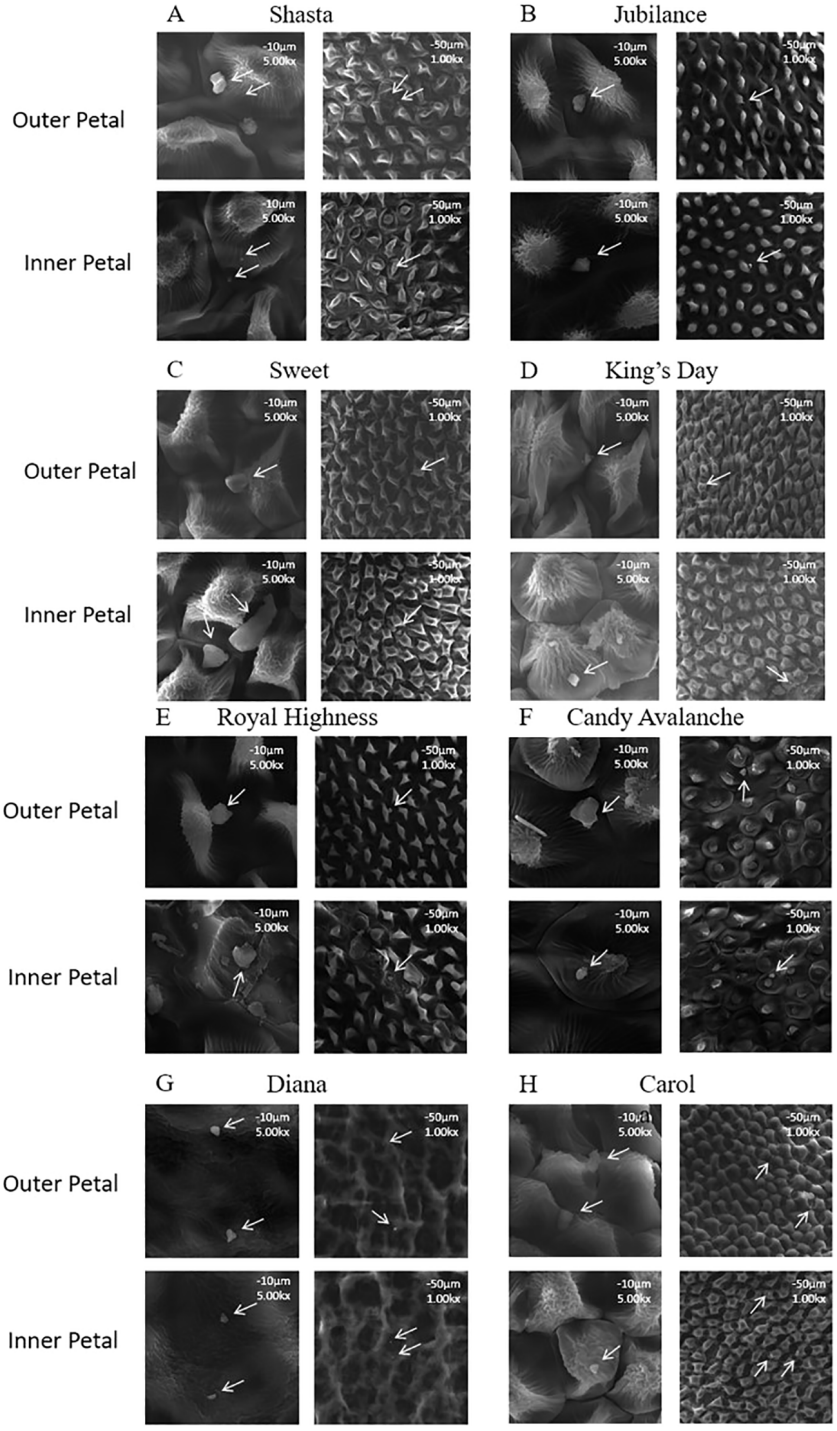
Micro-morphology of the wax crystal on rose petals **(A)** Mount Shasta; **(B)** Jubilance; **(C)** Sweet; **(D)** King’s Day; **(E)** Royal Highness; **(F)** Candy Avalanche; **(G)** Diana; **(H)** Carola; Wax crystal morphology of rose petal in full bloom was monitored at × 1000 (scale bars = 50 μm) and × 5000 (scale bars = 10 μm) magnification.

The wax crystals on the surfaces of the inner and outer petals of eight rose cultivars (**Figure 1**) were observed using scanning electron microscopy. There were no differences in the shapes and distribution density levels of the cuticular crystals between the inner and outer petals of the same variety (**Figure 2**). The wax crystal distributions of the eight rose varieties were sparse and basically granular, and only a few rod-shaped crystals were observed in ‘Sweet’.

### 3.2 Analysis of the wax composition and content on petals

**Figure 3:**
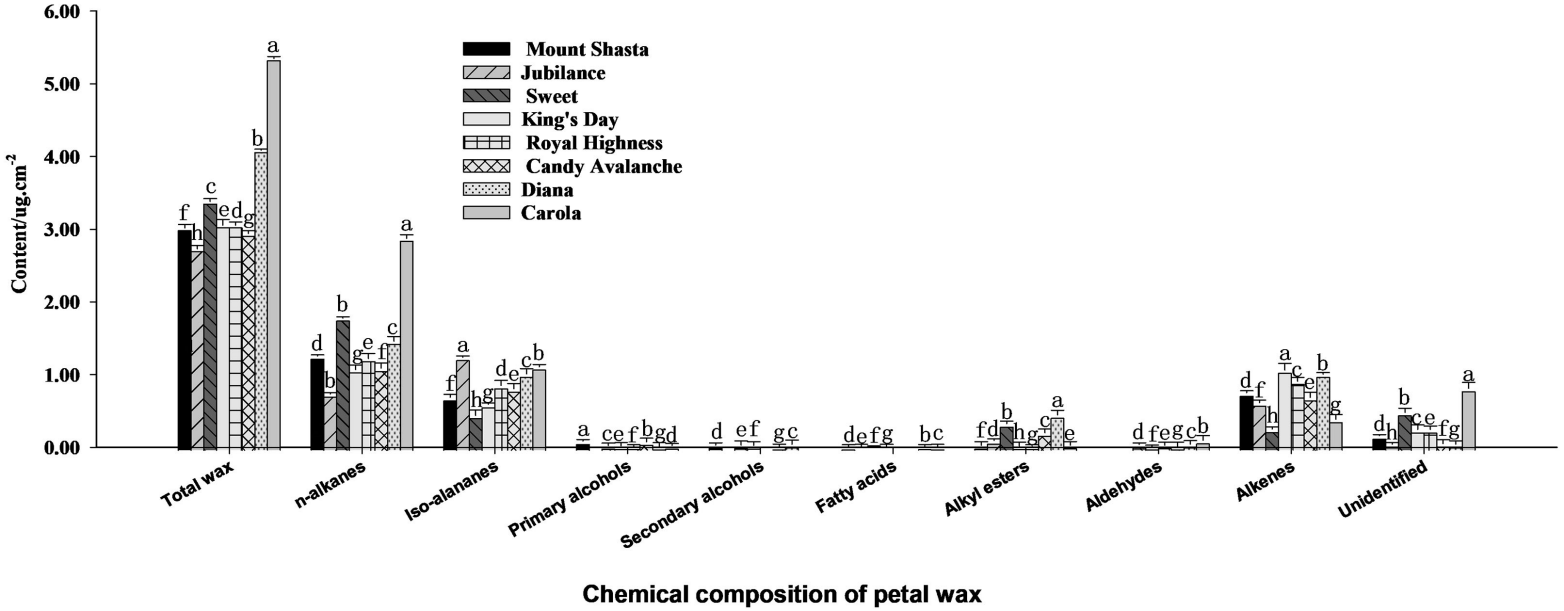
Analysis of contents of n-alkanes, iso-alananes, alkenes, alkylesters, primary alcohols, secondary alcohols, fatty acids, alkylesters and some unidentified compositions in full blooming rose petals Data are the mean ± SD of three independent replicates (n = 3). Lowercase letters indicate significantly differences between the eight rose cultivars at P < 0.05.

A GC-MS analysis of the chemical composition of cuticle waxes on rose petals, as shown in **Figure 3**, revealed that the waxes mainly includes n-alkanes, iso-alananes, alkenes, alkylesters, primary alcohols, secondary alcohols, fatty acids, alkylesters and some other unknown components. Those with the highest contents were n-alkanes, iso-alananes and alkenes. The total wax contents of ‘Carola’ and ‘Diana’ were the highest among the eight cultivars at 4.1 × 10^9^ μg·cm^−2^ and 5.3 × 10^9^ μg·cm^−2^, whereas the content of total wax contents of ‘Jubilance’ and ‘Candy Avalanche’ were the lowest at 2.7 × 10^9^ μg·cm^−2^ and 2.9 × 10^9^ μg·cm^−2^, respectively.

**Figure 4:**
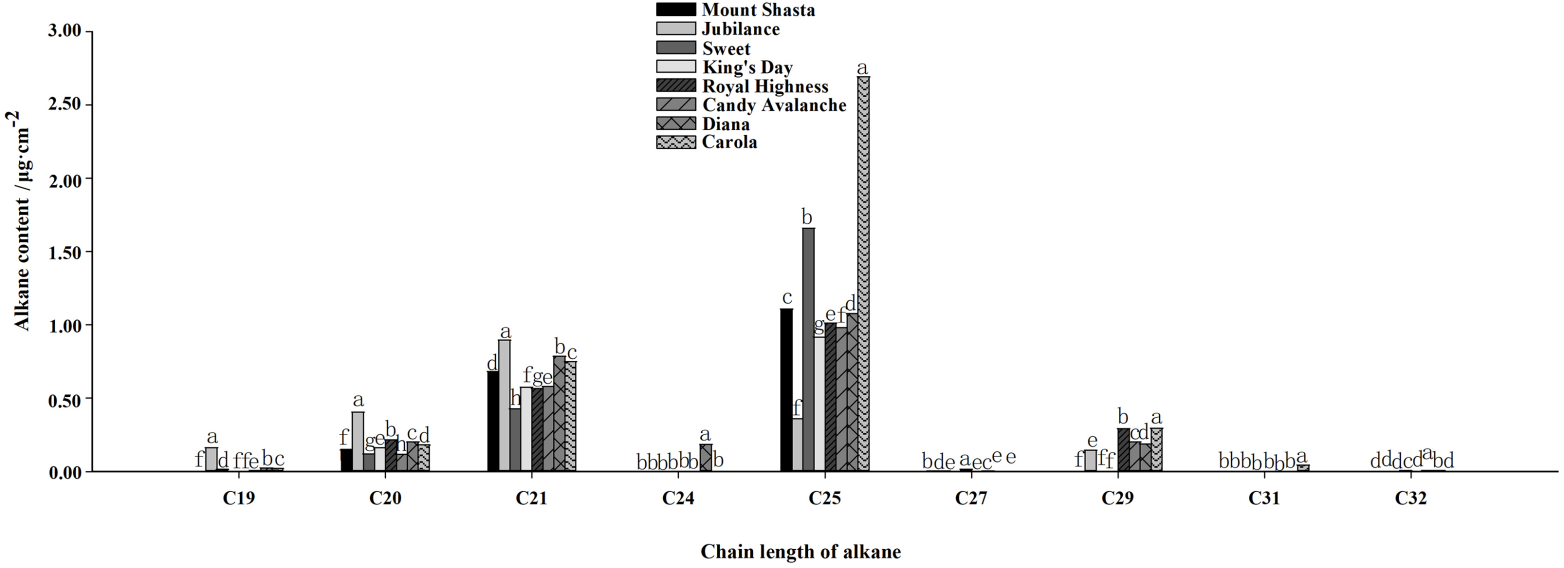
Analysis of alkane contents in full blooming rose petals. Data are the mean ± SD of three independent replicates (n = 3). Lowercase letters indicate significantly differences between the eight rose cultivars at P < 0.05.

**Figure 5:**
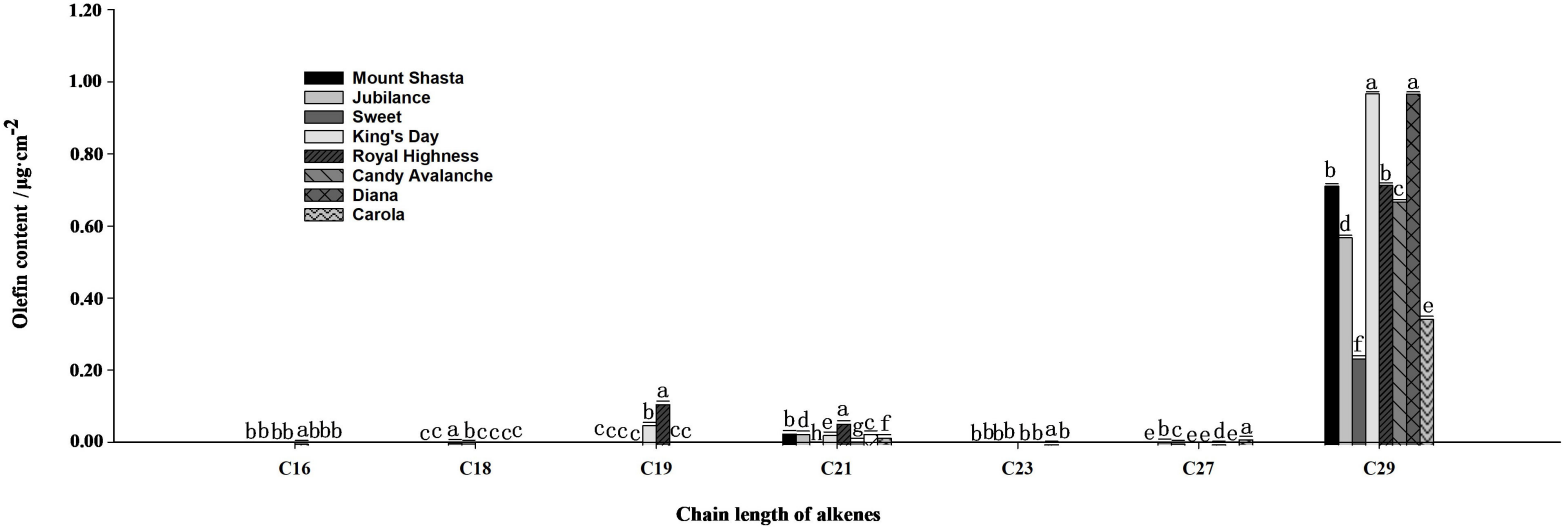
Analysis of olefin contents in full blooming rose petals. Data are the mean ± SD of three independent replicates (n = 3). Lowercase letters indicate significantly differences between the eight rose cultivars at P < 0.05.

The main compositions and contents of alkanes and olefins in rose petal waxes were also analyzed (**Figures 4**, **5**). The carbon chain lengths of alkanes ranged from C19 to C32, with C25 being the most predominant. The C25 content was the highest in ‘Carola’ (accounting for 21.82% of the total petal wax content) and lowest in ‘King’s Day’ (accounting for 9.18% of the total petal wax content). For alkenes, the lengths of carbon chains range from C16 to C29, with C29 being the most predominant. The C29 content was highest in ‘Diana’ (17.78% of the total petal wax) and lowest in ‘Sweet’ (12.14% of the total petal wax).

### 3.3 Water permeability analysis of petals

**Figure 6:**
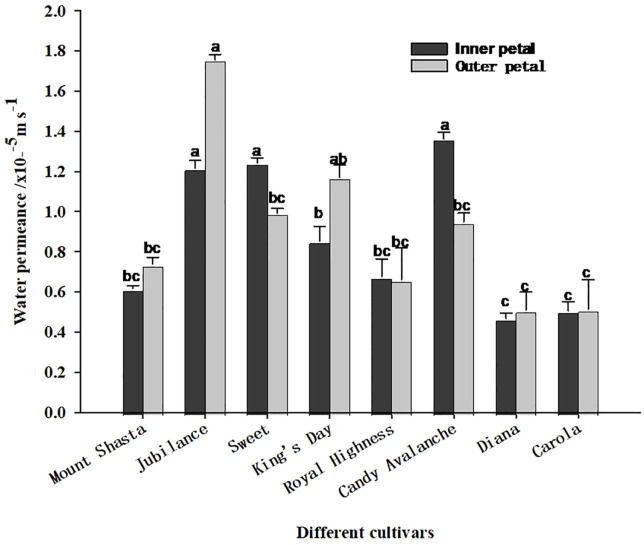
Water permeance of eight rose cultivars under well-watered and loss-watered. Data are the mean ± SD of three independent replicates (n = 3). Lowercase letters indicate significantly differences at P < 0.05 between the eight rose cultivars of inner petals or outer petals.

As shown in **Figure 6**, the water permeability levels of the inner and outer petals of the tested varieties of roses differed. Among the outer petals, those of ‘Jubilance’ had the greatest permeability, whereas among the inner petals, those of ‘King’s Day’ had the greatest permeability, followed by those of ‘Jubilance’ and ‘Sweet’. ‘Diana’ and ‘Carola’ had the lowest permeability levels for both inner and outer petals, which significantly differed from those of ‘Jubilance’ and ‘Candy Avalanche’. Thus, the petals of ‘Diana’ and ‘Carola’ formed the strongest barriers to water transpiration through the cuticle, whereas those of the ‘Jubilance’ and ‘Candy Avalanche’ formed the weakest barriers.

### 3.4 Vase life under water-loss stress

**Figure 7:**
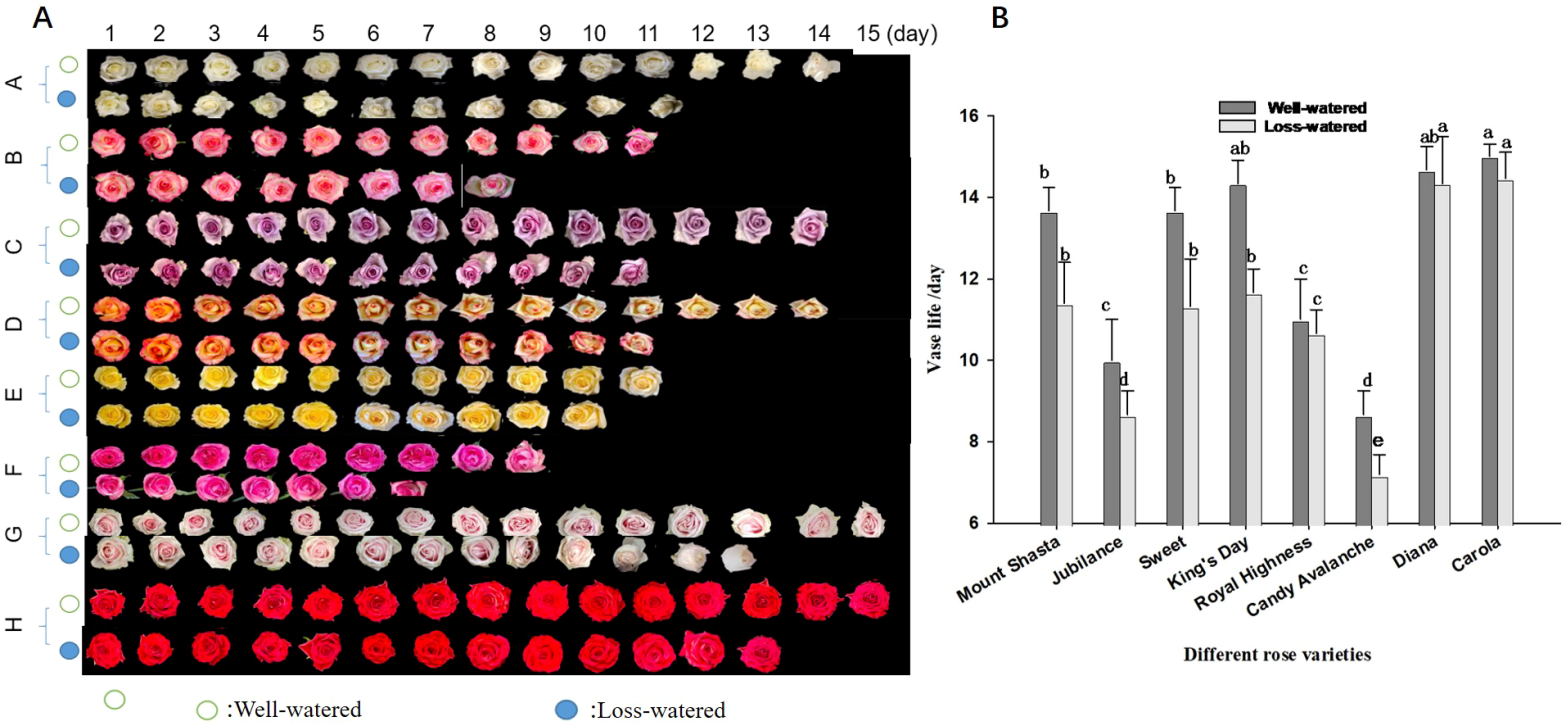
Effects of water loss stress on phenotype and vase life of eight rose cultivars. **(A)**: phenotype of eight rose cultivars (Mount Shasta(A), Jubilance(B); Sweet (C) King's Day (D); Royal Highness E; Candy Avalanche (F) Diana (G) Carola (H)); **(B)**: Comparison vase life of eight rose cultivars. Data are the mean ± SD of twenty independent replicates (n = 20). Lowercase letters indicate significantly differences between the eight roses cultivar under well-watered or loss-watered at P < 0.05.

The vase lives of the eight rose cultivars were compared under water-loss stress conditions (**Figures 7A, B**). ‘Carola’ and ‘Diana’ had the longest vase lives (15 d) under control conditions, whereas ‘Jubilance’ and ‘Candy Avalanche’ had the shortest vase lives (only 10d and 9d, respectively). Under water-loss stress conditions, the vase life of each cultivar decreased compared with under control conditions. However, the varieties having the shortest and longest vase lives did not change, namely the longest vase lives were found in ‘Carola’ and ‘Diana’ while ‘Jubilance’ and ‘Candy Avalanche’ had the shortest.

### 3.5 Relative water contents of petals of different rose cultivars

**Figure 8:**
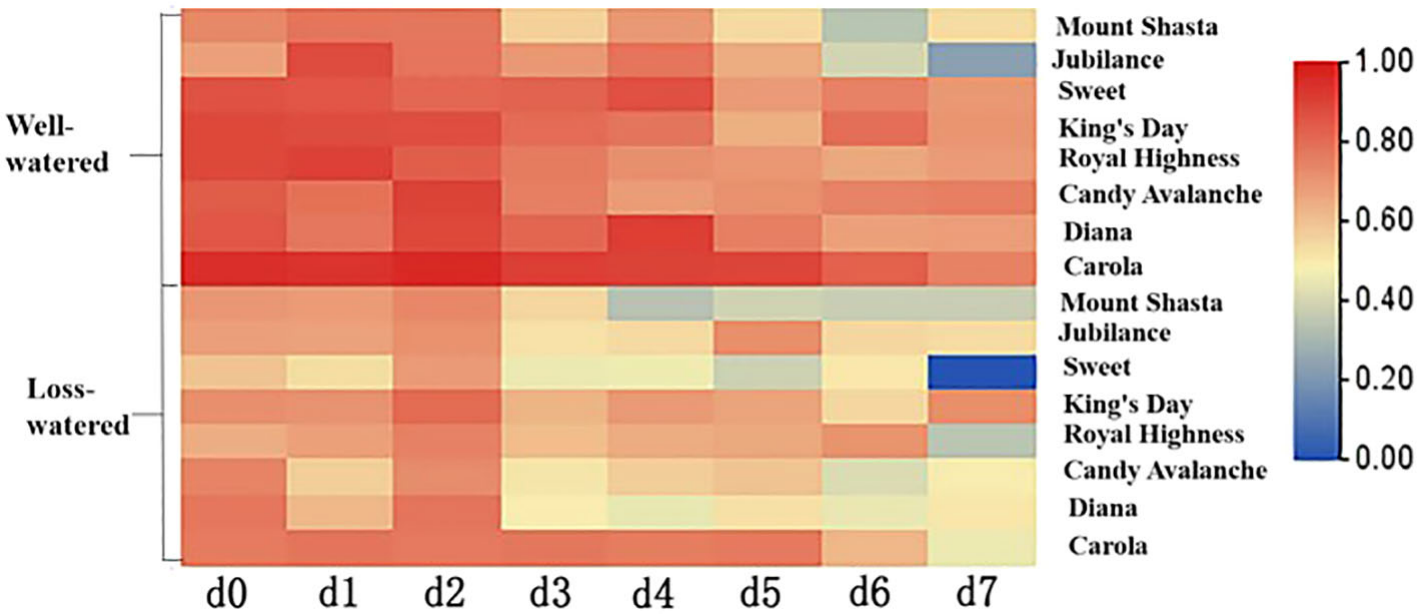
Heat map of relative water content of eight rose cultivars. Data are the mean of three independent replicates (n = 3).

The relative water contents of petals are shown in **Figure 8**. The relative water contents of all the cut roses showed decreasing trends from 0 to 7 d. On the 7th day, the relative water contents of ‘Diana’ and ‘Carola’ petals were the highest, being significantly greater than those of ‘Mount Shasta’, ‘Jubilance’, ‘Sweet’, ‘King’s Day’, ‘Royal Highness’ and ‘Candy Avalanche’. The petals of ‘Jubilance’ and ‘Candy Avalanche’ had the lowest relative water contents.

### 3.6 The relative electrical conductivity of petals

**Figure 9:**
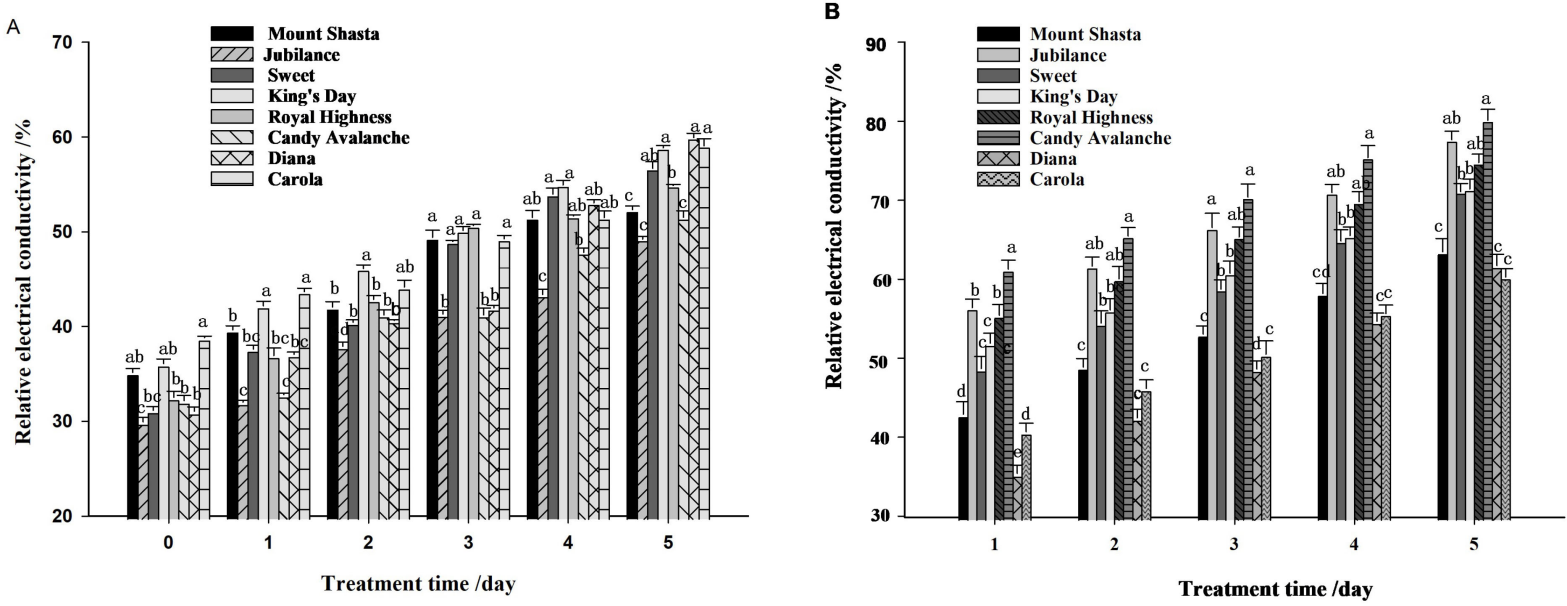
Relative electrical conductivity of eight rose cultivars. **(A)**: relative electrical conductivity under well-watered; **(B)**: relative electrical conductivity under loss-watered. Data are the mean ± SD of three independent replicates (n = 3). Lowercase letters indicate significantly differences at P < 0.05 between the eight rose cultivars at the same day.

The relative electrical conductivity of petals from cut roses increased under both control and water-loss stress conditions (**Figure 9A**), and water-loss stress induced the relative electrical conductivity (**Figure 9B**). On the 5th day ‘Candy Avalanche’ and ‘Jubilance’ had the highest relative conductivity levels, whereas ‘Diana’ and ‘Carola’ had the lowest relative conductivity levels under water-loss stress.

### 3.7 Pearson correlation analyses of vase life, total wax content, water permeability, relative electrical conductivity and relative water content under water-loss stress conditions

The correlation matrix of the five indicators, vase life, total wax content, water permeability, relative electrical conductivity and relative water content, under water-loss stress is shown in **Table 1**. The total wax content was positively correlated with vase life and negatively correlated with relative electrical conductivity, which was negatively correlated with vase life and positively correlated with water permeability.

**Table 1:** Pearson correlation analysis of Vase life, Water permeability, Total wax content, RWC and REC of eight rose species.

	Vase life	Water permeability	Total wax content	RWC	REC
**Vase life**	1				
**Water permeability**	-.684	1			
**Total wax content**	0.778^*^	-.518	1		
** RWC**	-.344	0.086	-.102	1	
** REC**	-.898^**^	0.799^*^	-.812^*^	0.37	1

## 4 Discussion

The e cuticular waxy layer of plants is the first barrier of self-protection. It can be divided into inner and outer layers, and the outer waxes can self-assemble to form unique wax crystal structures (Li et al., 2019). Here, the wax crystals of eight tested rose cultivars were found to be granular and only a few were rod-shaped. Su et al. (2019) showed that there are two types of wax crystals, plate-like and tubular, on the backs of flag leaves from three wheat varieties. The wax crystals on the petals of five lily cultivars are ellipsoidal (Zhao et al., 2023). The leaf cuticular wax crystals of alfalfa are lamellar (Wang et al., 2020). The structures of wax crystals are influenced by the chemical compositions of the waxes. Koch and Ensikat (2008) concluded that alkanes form smooth film-like wax layers without crystal structures, secondary alcohols or diketones mostly form tubular structures, and primary alcohols often form lamellar structures.

The chemical compositions of cuticular waxes vary greatly among different plant species. Those of *Brassica napus* and *Lycopersicon esculentum* are composed mainly of alkanes, whereas those of *Medicago sativa* and *Zea mays* are composed mainly of primary alcohols (Lee and Suh, 2015a). The wax of *Leymus chinensis* has diketone as the dominant component (Li et al., 2018), whereas the main components of pear cuticular waxes are alkanes, olefins, alcohols, aldehydes, esters and fatty acids (Mao et al., 2022). Zhao et al. (2023) found that the main components of petal cuticular waxes in five lily cultivars are alkanes and primary alcohols. Here, the main wax components of rose petals were alkanes, followed by alkenes and alcohols. For example, in ‘Carola’, alkanes, isoalkanes and alkenes accounted for 53.74%, 20.74% and 7.24% of the total wax components, respectively. The wax composition of the epidermis varies from genotype to genotype in the same species. A two-fold difference in total wax load has been observed in the stems of 40 *Arabidopsis* ecotypes (Rashotte et al., 1997). The contents of wax components in the leaves of seven epiphytic *Tillandsia* species differ significantly (Zheng and Li, 2023). In this study, ‘Carola’ and ‘Diana’ had the highest wax contents in petals, whereas ‘Jubilance’ and ‘Candy Avalanche’ had the lowest total wax contents. The composition of cuticular wax from the same tissue varies at different developmental stages. Bourgault et al. (2020) found that the levels of alkanes and olefins in maize leaves are highest in the leaf base, but the levels decreased by 70% from base to top along the developmental gradient of adult maize leaves. Cheng et al. (2021) investigated the types and contents of waxes in lily petals at the green bud and full flowering stages and found that the wax content increased along with the wax types in petals. The composition of the wax also changes significantly between flower opening and senescence in *Antirrhinum majus* L (Goodwin et al., 2003). These results suggest that cuticular wax biosynthesis is under fine control.

Cuticular wax has also been reported to play an essential role in resisting pathogens in plants on its stress resistance, especially drought resistance. Among the tested roses, ‘Diana’ and ‘Carola’, having the highest wax contents, had the longest vase lives, the highest relative water contents and the lowest relative electrical conductivity levels. The lowest wax contents of petals were found in ‘Jubilance’ and ‘Candy Avalanche’, which had the shortest vase lives and the lowest relative water contents under water-loss stress conditions. Li et al. (2019) also showed that the wax contents of drought-tolerant cultivars are 21.33% higher than those of drought-tolerant cultivars. Zhao et al. (2023) found that the petals of *Lilium* spp. that were strongly resistant to water-loss stress had high wax contents. Reports on other plants, such as oats, rice, sorghum, alfalfa and wheatgrass, have confirmed that a high cuticular wax content contributes to drought tolerance (Kunst and Samuels, 2003). Interestingly, studies in *Arabidopsis* suggest that alkanes in the waxes play important roles in drought resistance (Lee and Suh, 2022). In this research, ‘Carola’ and ‘Diana’, which had the strongest abilities to resist water-loss stress, had the highest contents of straight-chain alkanes and isoalkanes. In a study of lily cut flowers, alkanes were found to be the main wax components in the petals that were strongly resistant to water loss (Zhao et al., 2023). Studies on fruits also prove that peel cuticular wax contents affect water-loss resistance after postharvest. When litchi fruits were stored under dry, contents of n-alkanes, primary alcohols and very-long-chain fatty acids in cuticular waxes on the fruit surface changed significantly (Huang et al., 2023); a 2.3-fold increase in cuticular waxes in the browned pericarp was observed when compared with fresh fruit (Huang et al., 2022). The results of Chai et al. (2020) show that the total wax, particularly alkanes, in the peel of apple fruits is essential for storage and quality control.

Cuticular wax has also been reported to play an essential role in resisting pathogens in plants. For example, high wax formation in the ancestral grapevine correlates with reduced susceptibility to *E. necator* (Ge et al.,2023); an increased wax load on the goji leaves results in strengthened resistance to powdery mildew (Li et al., 2024); the cuticular wax of blueberry inhibited the growth of *Botrytis cinerea* (Liu et al., 2023). Pathogen is an important problem in the postharvest of roses, though it was not studied in this research, the results of this study are helpful for the future research in the area.

In this study, we found that the cuticular wax crystals of rose petals were mainly granular, and the wax components were mainly n-alkanes, iso-alananes and alkenes. The varieties having a strong tolerance to water-loss stress had high wax contents, whereas those sensitive to water-loss stress had low wax contents. The results of this study can be used for breeding new rose varieties tolerant to water-loss stress.

## Data Availability

The data analyzed in this study is subject to the following licenses/restrictions: All raw data can be provided if required. Requests to access these datasets should be directed to Jinfen Wen, wenjf888@163.com.
